# Subcutaneous Fluid Administration in Pediatric Sepsis With Challenging Intravenous Access

**DOI:** 10.7759/cureus.84581

**Published:** 2025-05-21

**Authors:** Mohamad Taha, Abdulla Elkhalili, Sharif Mohamed

**Affiliations:** 1 Anesthesiology, Texas A&M University College of Medicine, Bryan, USA; 2 College of Veterinary Medicine and Biomedical Sciences, Texas A&M University, League City, USA; 3 Anesthesiology, University of Texas Medical Branch, Galveston, USA

**Keywords:** dehydration, hypodermoclysis, intravenous access failure, pediatric sepsis, subcutaneous hydration

## Abstract

Subcutaneous intravenous (IV) fluid administration, also known as hypodermoclysis, is a method of delivering fluids or medications by injecting them into the subcutaneous tissue. This technique is rarely used in humans and is typically reserved for patients with severe dehydration and difficult IV access. In this case report, we describe a seven-week-old male infant with dehydration secondary to *Escherichia coli* pyelonephritis. After multiple failed IV attempts, even under general anesthesia, hypodermoclysis was performed. The patient subsequently achieved sufficient hydration, allowing for successful peripheral IV placement.

## Introduction

Urinary tract infections (UTIs) are a common cause of bacterial infection in infants, affecting up to 8% of children aged 1 month to 11 years, with a 30% recurrence rate within a year [1]. In the United States, they account for 1.5 million pediatric ambulatory visits annually [1]. *Escherichia coli *is responsible for 80%-90% of first UTIs in children and two-thirds of recurrent cases [2]. UTIs commonly present as fever, dysuria, and flank pain and may lead to long-term complications such as permanent kidney scarring [1]. Risk factors for UTIs in infants include a history of maternal UTI, uncircumcised males, anatomic abnormalities, and functional disorders such as vesicoureteral reflux [2,3].

In children hospitalized with pyelonephritis, intravenous (IV) fluids are recommended alongside antibiotics to optimize renal perfusion and support urine output [2]. Guidelines suggest administering normal saline for rapid intravascular volume repletion, with maintenance fluids typically consisting of 5% dextrose in normal saline or half-normal saline [2]. Peripheral IV fluid administration is the standard method for rehydration; however, securing IV access can be particularly challenging in severely dehydrated neonates and critically ill children. In such cases, alternative methods of fluid administration may be required. Hypodermoclysis, the subcutaneous administration of fluids, is a rarely used technique in humans but can support initial rehydration efforts when IV access is challenging, as demonstrated in this case. It is widely used in geriatric and palliative care settings as an easy and comfortable hydration method, allowing patients to meet their fluid needs without the discomfort of peripheral IV placement, while maintaining similar effectiveness [4]. Hypodermoclysis is relatively safe and straightforward, involving the insertion of a small needle into the subcutaneous tissues, typically in areas such as the thighs, abdomen, back, or arms [4]. This creates pockets of fluid within the subcutaneous space, allowing for gradual reabsorption into the bloodstream via diffusion and perfusion [4]. 

## Case presentation

A seven-week-old male infant initially presented to urgent care with a fever (101°F, 38.3°C), where he was diagnosed with a UTI with hematuria. The patient's mother did not report changes to his urine output, stools, feeding, behavior, or any signs of lethargy. The initial blood culture showed coagulase-negative *Staphylococcus *growth, and a repeat culture after 24 hours was negative. 

**Figure 1:**
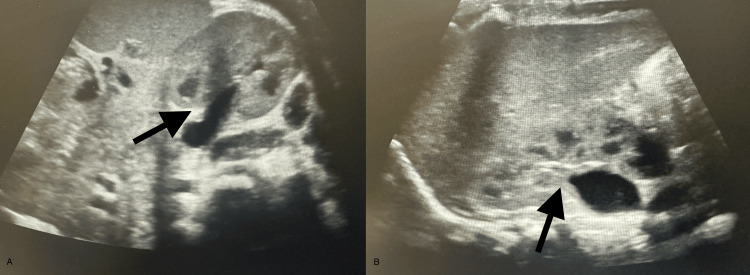
Ultrasound demonstrating bilateral grade 1 hydronephrosis in a seven-week-old infant with pyelonephritis

**Figure 2:**
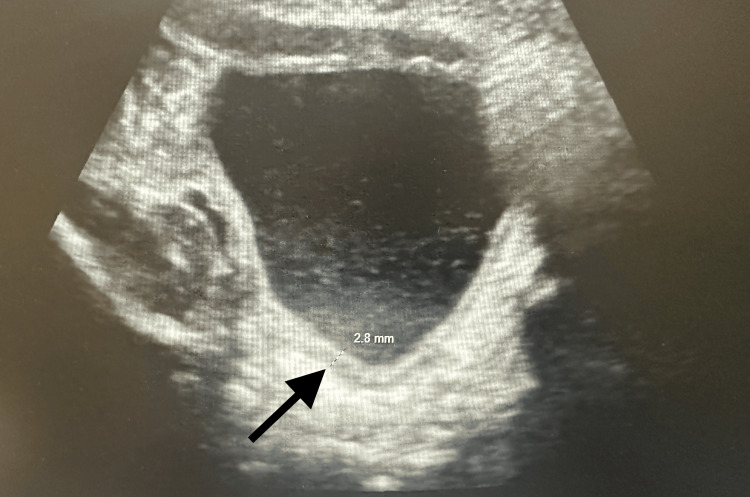
Ultrasound demonstrating a mildly thickened bladder wall with debris in a seven-week-old infant with pyelonephritis

On physical examination, the patient had a borderline capillary refill of 2-3 seconds, but no signs of acute distress. Initial laboratory results were notable for hyponatremia (127 mmol/L) and elevated inflammatory markers (C-reactive protein 6.2 mg/dL) (Table 1). Urinalysis findings consistent with a UTI, including cloudy urine, 176 white blood cells (WBCs), 500 leukocyte esterase, 2+ blood, 23 WBC clumps, and >100,000 CFU/mL *E. coli *(Table 2). Ultrasonography KUB was suggestive of bilateral grade 1 hydronephrosis (Figure 1) and a mildly thickened bladder wall with debris (Figure 2), confirming a diagnosis of *E. coli* pyelonephritis. Hematologic workup revealed mild anemia (hemoglobin 8.8 g/dL, hematocrit 27%), mild thrombocytosis (platelet count 459 × 10³/µL), and elevated segmented neutrophil (56%) and monocyte (15%) counts. The patient’s history is significant for perinatal exposure to HIV and possibly cocaine. Maternal viral load at delivery was unknown, and the patient had received prophylactic zidovudine, nevirapine, and lamivudine at birth. The patient also had two negative HIV quantitative tests at birth and at one month old. 

**Table 1:** Notable complete blood count results

Result	Value	Reference range
Sodium	127 mmol/L	132-145 mmol/L
C-reactive protein	6.2 mg/dL	<1 mg/dL
Hemoglobin	8.8 g/dL	10.5-14 g/dL
Hematocrit	27%	32%-42%
Platelets	459 × 10³/µL	133-320 × 10³/µL
Segmented neutrophils	56%	20%-46%
Monocytes	15%	0%-7%

**Table 2:** Abnormal urinalysis results

Result	Value	Reference range
White blood cells	176	0-5/high-power field
Leukocyte esterase	500	Negative
Blood	2+	Negative
White blood cell clumps	23	Negative
E. coli	>100,000 CFU/mL	Negative

Upon arrival at the emergency department, repeated attempts at peripheral IV placement were unsuccessful despite efforts by NICU nurses and pediatric anesthesiologists. A jugular central line placement was attempted under general anesthesia, which was induced using inhaled anesthetic agents, but was also unsuccessful due to severe dehydration. Given the continued difficulty in obtaining IV access, a single subcutaneous injection site was initiated in the right lower quadrant of the abdomen using a hypodermic needle, allowing for the administration of 100 mL of normal saline (0.9% NaCl) over a 20-minute period. Once an intraosseous kit became available, an intraosseous line was placed in the left tibia for additional fluid resuscitation. This ultimately enabled sufficient rehydration to achieve successful peripheral IV placement in the left neck and left arm for ongoing fluid administration.

Throughout his hospitalization, the patient remained hemodynamically stable, with resolution of fever and tolerance of oral intake. Electrolyte repletion was achieved with 0.9% NaCl infusion, and IV fluids were discontinued prior to discharge. The patient’s UTI was treated with IV ceftriaxone and later transitioned to oral amoxicillin-clavulanate upon discharge.

## Discussion

Sepsis in newborns remains a leading cause of mortality, accounting for up to 15% of infant deaths worldwide [5]. Rapid recognition and aggressive treatment are required to improve clinical outcomes. This life-threatening condition is characterized by a bacterial, fungal, or viral infection within the blood, triggering a systemic inflammatory response and potentially resulting in hemodynamic instability and multi-organ failure [6,7]. The most frequent bacterial causes include Group B *Streptococcus* and *E. coli* in early-onset cases, while *Staphylococcus* species are more common in late-onset sepsis [8]. Due to the immaturity of immune defenses, newborns are particularly vulnerable, making early diagnosis critical [9]. The clinical signs of sepsis in a newborn are very often nonspecific but may include fever, lethargy, irritability, poor feeding, and respiratory distress [10]. Proper fluid hydration is a key aspect in sepsis management, essential for stabilizing a septic newborn; however, dehydration remains a significant challenge in this population due to difficult IV access [11]. Traditional IV therapies are often challenging due to infants’ fragile vasculature, further necessitating alternative methods such as intraosseous access or subcutaneous fluid administration [11,12].

**Figure 3:**
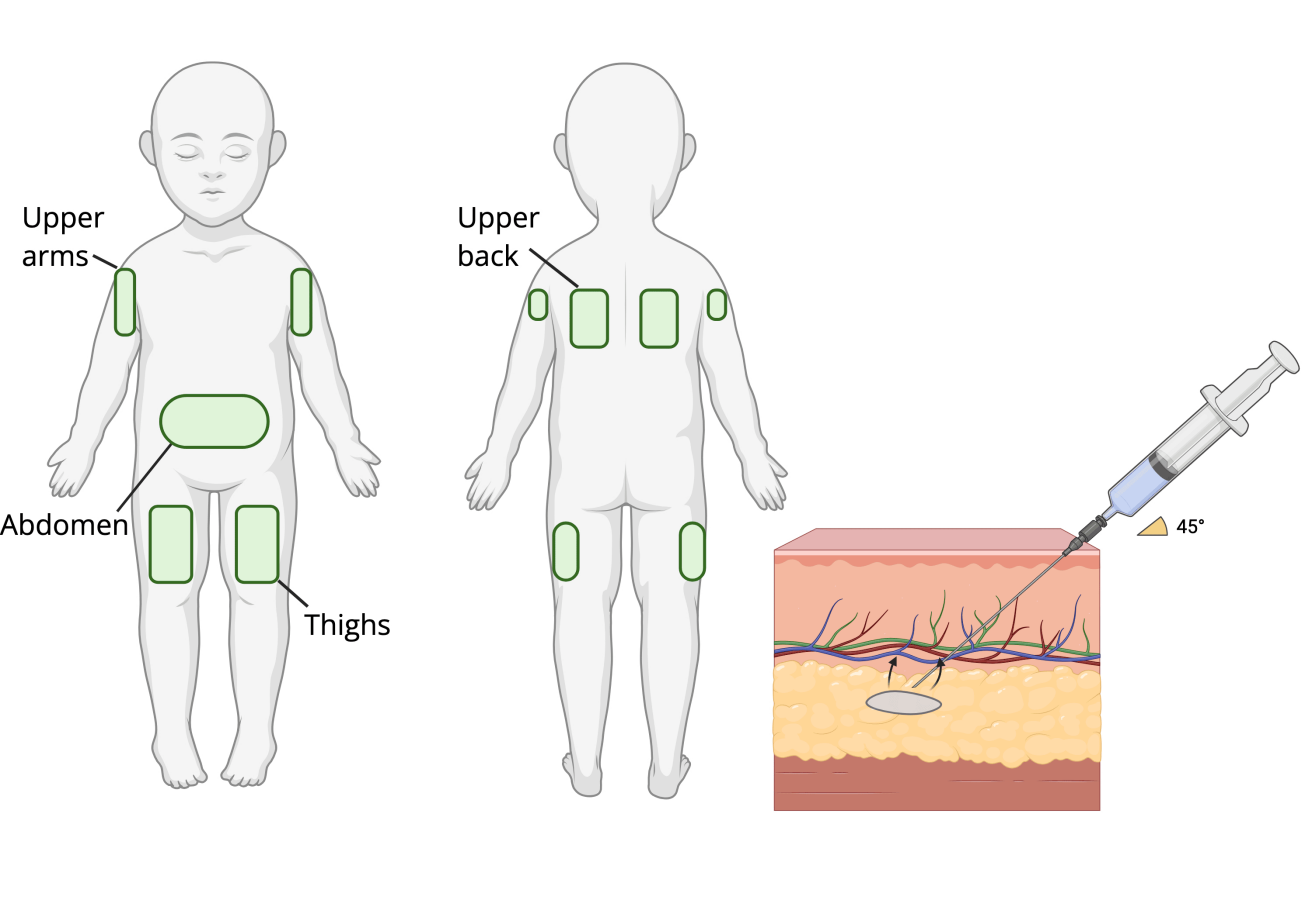
Optimal sites for subcutaneous fluid injection Subcutaneous fluid administration creates temporary pockets in the interstitial space, allowing for gradual absorption into the circulation [4,14]. Image Credit: This is an original image created by author Mohamad Taha.

Hypodermoclysis is a common method of hydration and medicinal delivery that has been used extensively for geriatric patients and in palliative care settings [4,13]. This technique involves the controlled infusion of fluids into the subcutaneous tissue via a small needle, ensuring a gradual yet thorough absorption of fluid into the bloodstream [4,14]. Typically, a 22- to 24-gauge needle is inserted at a 45-degree angle into regions with sufficient subcutaneous tissue, such as the abdomen, thighs, and interscapular region (Figure 3) [4]. Once in position, the needle is secured with a transparent dressing or bandage, similar to an IV catheter, and then connected to the fluid source [4]. Fluids can be administered via gravity or a pump, with careful monitoring to prevent local edema [4]. Butterfly needles (winged infusion sets) are usually preferred over angiocatheters for short-term infusions due to their easier maneuverability and better control; however, they typically have a shorter lifespan [13]. Subcutaneous fluid administration has shown several advantages compared to traditional IV therapy, including procedural simplicity and lower overall cost of infusion [4,15]. Although typically minimal, subcutaneous fluid administration may pose potential health complications to patients, such as edema, erythema, and mild pain at the infusion site [13,15].

Unfortunately, research on the use of hypodermoclysis in pediatric populations remains limited, though some notable studies have been conducted. Small-scale trials have reported that hypodermoclysis can be a feasible alternative for mild-to-moderate dehydration in pediatric patients [16]. The technique has demonstrated considerable success among palliative care and geriatric patients, offering low rates of failure and favorable safety outcomes, including minimal complications after injection [17]. These results suggest that hypodermoclysis could potentially be applied in pediatric care. One research study indicates that the use of recombinant human hyaluronidase, an enzyme that improves fluid dispersion, may improve absorption rates in infants and young children when using hypodermoclysis [16]. Other studies have clarified the uncertainty of this technique’s efficacy. Research suggests that neonates and infants hold a higher percentage of total body water, which may influence the absorption and distribution of fluids [18]. Additionally, their metabolic rates are higher than those of adults, leading to faster fluid consumption and potentially different hydration needs [19,20]. The present case highlights the challenges associated with IV placement in a septic infant with *E. coli* pyelonephritis. Given the need for rapid rehydration, subcutaneous fluid administration was utilized for initial hydration. This allowed for sufficient fluid resuscitation until successful peripheral IV placement was achieved. 

## Conclusions

This case underscores the importance of early IV placement in dehydrated pediatric patients to prevent progression to severe dehydration and the potential need for general anesthesia. While peripheral IV fluid administration remains the standard, securing IV access can be challenging in critically ill children. As demonstrated in this case, hypodermoclysis can supplement rehydration efforts when IV access proves difficult, providing fluid resuscitation until a peripheral IV can be established. Further investigation into the safety and efficacy of hypodermoclysis in neonates and infants is necessary, as optimizing hydration strategies is crucial for improving outcomes, particularly in patients with sepsis or severe bacterial infections such as pyelonephritis.
